# Neglected zoonotic agents in cattle abortion: tackling the difficult to grow bacteria

**DOI:** 10.1186/s12917-017-1294-y

**Published:** 2017-12-02

**Authors:** Sara Vidal, Kristel Kegler, Gilbert Greub, Sebastien Aeby, Nicole Borel, Mark P. Dagleish, Horst Posthaus, Vincent Perreten, Sabrina Rodriguez-Campos

**Affiliations:** 1 Institute of Veterinary Bacteriology, Vetsuisse Faculty, University of Bern, Laenggassstrasse 122, CH-3012 Bern, Switzerland; 2 Graduate School for Cellular and Biomedical Sciences, Theodor Kocher Institute, University of Bern, Freiestrasse 1, CH-3001 Bern, Switzerland; 3 Institute of Animal Pathology, Vetsuisse Faculty, University of Bern, Laenggassstrasse 122, CH-3012 Bern, Switzerland; 4 Institute of Microbiology, University Hospital Center and University of Lausanne, Bugnon 48, CH-1011 Lausanne, Switzerland; 5 Institute of Veterinary Pathology, Vetsuisse Faculty, University of Zurich, Winterthurerstrasse 270, CH – 8057 Zurich, Switzerland; 6 Moredun Research Institute, Pentlands Science Park, Bush Loan, Penicuik, Edinburgh, Scotland EH26 0PZ UK

**Keywords:** *Coxiella burnetii*, *Chlamydiales*, *Leptospira* spp., Bovine abortion, Zoonosis

## Abstract

**Background:**

*Coxiella burnetii*, *Chlamydia abortus* and *Leptospira* spp. are difficult to grow bacteria that play a role in bovine abortion, but their diagnosis is hampered by their obligate intracellular lifestyle (*C. burnetii*, *C. abortus*) or their lability (*Leptospira* spp.). Their importance is based on the contagious spread in food-producing animals, but also as zoonotic agents. In Switzerland, first-line routine bacteriological diagnostics in cattle abortions is regulated by national law and includes only basic screening by staining for *C. burnetii* due to the high costs associated with extended spectrum analysis. The aim of this study was to assess the true occurrence of these zoonotic pathogens in 249 cases of bovine abortion in Switzerland by serology (ELISA for anti-*C. burnetii* and *C. abortus* antibodies and microscopic agglutination test for anti-*Leptospira* spp. antibodies), molecular methods (real-time PCR and sequencing of PCR products of *Chlamydiales-*positive cases), Stamp’s modification of the Ziehl-Neelsen (mod-ZN) stain and, upon availability of material, by histology and immunohistochemistry (IHC).

**Results:**

After seroanalysis the prevalence was 15.9% for *C. burnetii*, 38.5% for *C. abortus* and 21.4% for *Leptospira* spp. By real-time PCR 12.1% and 16.9% of the cases were positive for *C. burnetii* and *Chlamydiales*, respectively, but only 2.4% were positive for *C. burnetii* or *Chlamydiales* by mod-ZN stain. Sequencing of PCR products of *Chlamydiales-*positive cases revealed *C. abortus* in 10% of cases and the presence of a mix of *Chlamydiales*-related bacteria in 5.2% of cases. Pathogenic *Leptospira* spp. were detected in 5.6% of cases. Inflammatory lesions were present histologically in all available samples which were real-time PCR-positive for *Chlamydiales* and *Leptospira* spp. One of 12 real-time PCR-positive cases for *C. burnetii* was devoid of histological lesions. None of the pathogens could be detected by IHC.

**Conclusion:**

Molecular detection by real-time PCR complemented by histopathological analysis is recommended to improve definitive diagnosis of bovine abortion cases and determine a more accurate prevalence of these zoonotic pathogens.

**Electronic supplementary material:**

The online version of this article (10.1186/s12917-017-1294-y) contains supplementary material, which is available to authorized users.

## Background

Abortion in dairy cattle is one of the major causes of economic loss in the livestock industry [1] and three of the bacterial agents that are implicated in bovine abortion during mid- to late-gestation are the difficult to grow: *Coxiella burnetii*, *Chlamydia abortus* and pathogenic *Leptospira* spp*.* Their importance is based on not only in the economic loss in animal production but also in their zoonotic risk [2–4].

*C. abortus* and *C. burnetii* are obligate intracellular Gram-negative bacteria. *C. abortus*, the causative agent of ovine enzootic abortion, may also lead to reproductive disorders in large ruminants [2, 5] and is known to cause spontaneous abortion in pregnant women [5, 6]. Other members of the families *Chlamydiaceae*, *Parachlamydiaceae* and *Waddliaceae* have also been found to play a possible role in abortion in ruminants as well as in humans [7–11]. *C. burnetii* has a wide host range, including domestic and wild animals. Infection in most animals is subclinical or presents with non-specific clinical signs, whereas ruminants, the main reservoir of infection, may present with late abortion and stillbirths; moreover, *C. burnetii* might be associated with metritis and infertility in cattle [3, 12–18]. Human infection with *C. burnetii* is known as Q fever and can lead to miscarriage in women [19–21]. Leptospirosis is caused by Gram-negative, pathogenic spirochetes of the genus Leptospira that is divided in more than 250 pathogenic serovars worldwide, which are classified into 25 serogroups on the basis of their serological phenotype. In cattle, leptospirosis is mainly associated with reproductive problems including infertility, low conception rate, abortion, stillbirths and weak offspring [22–24]. Cattle are considered to be the maintenance host of serovar Hardjo resulting in a high degree of subclinical infections [25]. Human leptospirosis occurs worldwide, is transmitted via direct or indirect contact with urine from infected animals and is due mostly to recreational and occupational activities [26–28]. Numerous outbreaks of leptospirosis worldwide have been also associated with heavy rainfall and flooding [29, 30]. Abortion in women due to leptospirosis may occur if infection takes place during pregnancy [31, 32].

Given the numerous possible etiologies of abortion in ruminants and the high cost of definitive diagnosis, a finance-limited investigation is performed usually, and the causative agent often remains undetermined [33]. Of the three cattle abortifacient pathogens discussed, only investigation of *C. burnetii* is legally regulated in Switzerland requiring Stamp’s modification of the Ziehl-Neelsen (mod-ZN) stain [34] of tissue smears [Ordinance on Epizootic Diseases (TSV) SR.916.401; Article 129].

According to the epizootics database of the Swiss Federal Food Safety and Veterinary Office (InfoSM www.infosm.blv.admin.ch, consulted on 02/08/2017), 676 cases of coxiellosis in cattle were reported from 2006 to 2016. Although pathogenic *Leptospira* spp. and *C. abortus* are not included in routine bovine abortion diagnostics and, moreover, *Leptospira interrogans* serovar Hardjo is exempt from mandatory notification in cattle, 43 cases of leptospirosis and 23 cases of chlamydiosis in cattle were reported in the same time frame.

In this study, the recommended mod-ZN method was complemented with serology, molecular methods, histology and immunohistochemistry to determine the degree of underestimation of the three abortifacient pathogens *C. burnetii*, *C. abortus* and pathogenic *Leptospira* spp. in bovine abortion in Switzerland.

## Methods

### Collection of samples

Samples from 249 cases of bovine abortion from different cantons of Switzerland were collected from October 2012 to October 2015 [Bern (*n* = 213), Vaud (*n* = 7), Fribourg (*n* = 6), Jura (*n* = 6), Solothurn (*n* = 6), Aargau (*n* = 2), Basel-Land (*n* = 2), Neuchâtel (*n* = 2), Valais (*n* = 2), Zurich (*n* = 2) and Luzern (*n* = 1)]. The 249 cases comprised 242 placentas, 57 fetal abomasal contents and 182 maternal sera submitted for routine abortion diagnostics. Placenta from a healthy calf was included as a negative control.

### Stamp’s modification of the Ziehl-Neelsen stain

Smears of placentas, abomasal contents (*n* = 299) and the negative control placenta were subjected to mod-ZN staining [32] and examined by light microscopy. *Chlamydia*-positive placental tissue was included as positive control in every stain. The sample was considered positive for *Chlamydiales* and/or *C. burnetii* when intracytoplasmic red-stained coccobacilli appeared in clumps against a blue background. The technique does not allow a differentiation between *Chlamydiales* and *C. burnetii*.

### Serological studies

The 182 maternal sera were tested for antibodies against *C. burnetii* and *C. abortus* using the commercial CHEKIT® Q fever antibody ELISA Test Kit and CHEKIT® *C. abortus* Antibody Test Kit (IDEXX, Liebefeld-Bern, Switzerland) according to the manufacturer’s instructions. The results were expressed as S/P values and derived from the ratio between optical density (OD) of the sample (S) and the OD of positive control (P) included in the kits. IDEXX state an S/P  ≥ 40% is considered positive, an S/P  < 30% is considered negative, and S/P values between these are considered suspect positive.

The serological detection of antibodies against *Leptospira* spp. was performed by microscopic agglutination test (MAT) (Manual of Diagnostic Tests and Vaccines for Terrestrial Animals of the Ordinance of Epizootic Diseases [22]). Twelve serovars were included in the test panel: Australis, Autumnalis, Ballum, Bataviae, Bratislava, Canicola, Grippotyphosa, Hardjo, Icterohaemorrhagiae, Pomona, Sejroe and Tarassovi (Additional file 1: Table S1). Sera were screened initially for agglutination at a dilution of 1:100 in sterile 0.85% NaCl. Reactive sera were titrated in two-fold serial dilutions to determine the end-point titer defined as the dilution at which at least 50% agglutination occurs. In every serological analysis negative and positive control sera were included as controls.

### DNA extraction and molecular studies

For the extraction of total genomic DNA 2 g of placenta or 2 mL of fetal abomasal content were suspended in 5 mL 0.85% NaCl in an IKA® DT-20 tube [35] and homogenized twice for 30 s at 6000 rpm, using the IKA ULTRA-TURRAX® tube drive. Subsequently, 500 μL of the homogenates were used for DNA extraction using QIAamp Mini Kit (Qiagen, Hombrechtikon, Switzerland). Fluorometric quantification of DNA was performed by Quantus™ Fluorometer (Promega, Dübendorf, Switzerland).

Real-time PCR targeting the IS*1111* of *C. burnetii* was performed according to Howe et al. [36]: IS1111-F801: 5′ AATTTCATCGTTCCCGGCAG 3′; IS1111-R901: 5′ GCCGCGTTTACTAATCCCCA 3′; probe IS1111-p822S-MGB: 5′ 6FAM-TGTCGGCGTTTATTGG–MGBNFQ 3’. PCR was performed in a total volume of 25 μL, 1X final concentration of TaqMan Universal PCR Master Mix (Applied Biosystems, Foster City, CA, USA), 1 μM of each primer, 80 nM of the probe, 0.5X of internal positive control (IPC) Template, 0.5X IPC Mix and 2.5 μL of the template. The following conditions were applied: 94 °C for 2 min, 40 cycles of 94 °C for 15 s and 60 °C for 30 s. Amplification was performed in duplicate on the TaqMan 7500 Fast Real-time PCR System (Applied Biosystems, Zug, Switzerland). As positive and negative controls *C. burnetii* DNA and water were used, respectively. Samples were considered positive when showing an exponential amplification curve up to cycle 39 in both replicates.

A pan-*Chlamydiales* real-time PCR targeting the *Chlamydiales* 16S rDNA was performed according to Lienard et al. [37]: panCh16F2: 5’ CCGCCAACACTGGGACT 3’; panCh16R2: 5’ GGAGTTAGCCGGTGCTTCTTTAC 3’; probe panCh16S: 5’ 6FAM-CTACGGGAGGCTGCAGTCGAGAATC-BHQ1 3’. PCR assays were performed in 20 μL, with iTaq Supermix with ROX (Bio-Rad, Reinach, Switzerland), 0.1 μM concentrations of each primer (Eurogentec, Seraing, Belgium), a 0.1 μM concentration of probe (Eurogentec), molecular-biology-grade water (Sigma-Aldrich, Buchs, Switzerland) and 5 μL of DNA sample. The cycling conditions were 3 min at 95 °C, followed by 50 cycles of 15 s at 95 °C, 15 s at 67 °C and 15 s at 72 °C. Samples were tested in duplicate using a StepOnePlus™ Real-time PCR System (Applied Biosystems, Foster City, CA, USA). As positive and negative controls *C. abortus* DNA and water were used, respectively. Samples were considered positive when showing an exponential amplification curve up to cycle 40 in both replicates. Samples exhibiting a cycle threshold (Ct) of ≤35 cycles were sequenced using specifically designed internal sequencing primers as described by Lienard et al. [37]. Obtained sequences were edited and analyzed by BLAST on the NCBI website (http://www.ncbi.nlm.nih.gov).

Real-time PCR targeting the *lipL*32 gene of *Leptospira* spp. was performed using primers and probe described by Villumsen et al. [38]: LipL32-F: 5′ AGAGGTCTTTACAGAATTTCTTTCACTACCT 3′; LipL32-R: 5′ TGGGAAAAGCAGACCAACAGA 3′; probe LipL32-P: 5' 6FAM-AAGTGAAAGGATCTTTCGTTGC-MGBNFQ 3'. PCR was performed in a total volume of 25 μL, 1X final concentration of TaqMan Universal PCR Master Mix, 1 μM of each primer, 80 nM of the probe, 0.5X of IPC Template and 0.5X IPC Mix and 2.5 μL of the template. The following conditions were applied: 94 °C for 2 min, 45 cycles of 94 °C for 15 s and 60 °C for 30 s using the TaqMan 7500 Fast Real-time PCR System. DNA of *Leptospira* spp. serovar Icterohaemorrhagiae strain RGA and water were used as positive and negative controls, respectively. Samples were considered positive when showing an exponential amplification curve up to cycle 40 in both replicates.

### Histopathology

To assess the significance of the molecular analysis, all cases with real-time PCR-positive results were examined histopathologically and by IHC (*n* = 32) when the placental tissue was available and was not severely autolytic. Selected samples of placenta were fixed in buffered formalin (10%), processed routinely through graded alcohols and embedded in paraffin-wax. Sections (4 μm) were mounted on Thermo Scientific™ SuperFrost Plus© (Braunschweig, Germany) slides and stained with hematoxylin and eosin (HE) for histological evaluation.

### Antibodies

For immunohistochemistry, mouse monoclonal anti-*Coxiella burnetii* antibody (clone 3.13, Squarix GmbH, Marl, Germany) diluted 1:500 in Tris-buffered saline (TBS), an anti-*Chlamydiaceae*-specific antibody directed against the chlamydial lipopolysaccharide (LPS, Clone ACI-P, Progen, Heidelberg, Germany) diluted 1:200 in antibody diluent (Glostrup, Denmark) and a rabbit polyclonal anti-LipL32 antibody (kindly provided by Dr. Jarlath Nally) diluted 1:1000 in phosphate-buffered saline (PBS) for detection of pathogenic *Leptospira* spp. were used.

### Immunohistochemistry (IHC)

All real-time PCR-positive cases for *C. burnetii* (*n* = 13), *Chlamydiales* (*n* = 14) and *Leptospira spp.* (*n* = 5) were subjected to immunohistochemistry when tissue was available and not autolytic.

Briefly, for all three antibodies 4 μm thick sections were deparaffinized and rehydrated through graded alcohols.

For *C. burnetii*, sections were immersed in 3% H_2_O_2_ in methanol (v/v) for 20 min to quench endogenous tissue peroxidases. Non-specific antibody binding was blocked with 25% normal goat serum (NGS, Vector Laboratories, Peterborough, UK) in TBS for 30 min and incubated with the primary antibody overnight at 4 °C. Visualization of the bound anti-*C. burnetii* primary antibody was by EnVision Kit (goat anti-mouse horseradish peroxidase conjugate, DakoCytomation, Ely, UK) according to the manufacturer’s instructions followed by addition of the chromogen 3-amino, 9-ethyl-carbazole (AEC, Vector Laboratories, Peterborough, United Kingdom) for 10 min.

*Chlamydiaceae* immunohistochemistry was performed as described by Borel et al. [39] using the detection kit Dako ChemMate (Dako, Glostrup, Denmark).

Immunohistochemistry for pathogenic *Leptospira* spp. was performed using the avidin-biotin-peroxidase complex (ABC) method. Sections were treated with 0.5% H_2_O_2_ in methanol (v/v) for 30 min to block endogenous peroxidase, heated in sodium-citrate buffer for 30 min in the microwave for antigen retrieval, incubated with 20% goat serum for 30 min, then incubated with the respective primary antibody overnight at 4 °C. Biotinylated goat-anti-rabbit IgG (BA-1000) diluted 1:200 in PBS (Vector Laboratories, Burlingame, CA, USA) was used as secondary antibody with incubation time of 60 min. Colour development was with 3,3′-diaminobenzidine tetrahydrochloride (DAB) with H_2_O_2_ (0.03%, pH 7.2) for 5 min.

Sections immunolabeled with the respective primary antibodies against *Chlamydiaceae*, *C. burnetii* and pathogenic *Leptospira* spp. were all counterstained with hematoxylin prior to mounting in an appropriate mountant.

Four qPCR-negative cases for all three agents were included as negative controls. For *C. burnetii* and pathogenic *Leptospira* spp., primary antibodies were substituted with an isotype matched normal mouse IgG antibody or normal rabbit IgG (1:3000; R4505; Sigma Aldrich, Taufkirchen, Germany), respectively, as method negative control preparations.

Sections of intestinal tissue from gnotobiotic piglets experimentally infected with porcine *Chlamydia suis* strain S45/6, *C. burnetii*-positive sheep and human placentas and hamster kidney infected with *L. interrogans* serovar Hardjo JB191 were included as positive controls.

### Statistical analysis

We calculated the degree of agreement between the serological and the molecular tests for *C. burnetii*, *C. abortus* and *Leptospira* spp. carried out in 182 cases using Cohen’s kappa (κ) coefficient with 95% of CIs with the online software GraphPad (http://graphpad.com/quickcalcs/kappa2). Standard cutoffs were used to define poor (κ < 0.40), fair (κ = 0.41–0.60), good (κ = 0.61–0.80) and very good agreement (κ ≥ 0.80). The techniques that do not allow for detection of a specific pathogen or yielded only negative results were not included in the comparison.

## Results

### Stamp’s modification of the Ziehl-Neelsen stain

Of the 299 tissue smears, 10 placental smears and two of abomasal contents were positive as denoted by the presence of red intracytoplasmic organisms consistent with coccobacilli. One of the positive placenta and abomasal content samples were from the same case (Additional file 2: Table S2).

### Detection by serological analysis

Of the 182 sera tested, 29 (15.9%) were positive for *C. burnetii* and two (1.1%) were suspect positive. Chlamydial antibodies were detected in 70 (38.5%) of the 182 sera and 23 (12.6%) sera were suspect positive. The prevalence of antibodies against *Leptospira* spp. was 39/182 (21.4%), with 21 (11.5%) sera being positive for at least two serovars. Serovar Hardjo was the most frequent (31/39) followed by serovar Sejroe (14/39). Yet, 12 sera were positive for both serovars with 10 sera showing a higher titer for Hardjo and, hence, indicating that the latter is the causative serovar. Six cases were positive for serovar Australis (Table 1).

**Table 1:** Positive samples by microscopic agglutination test for the 12 tested serovars of *Leptospira* spp.

Sample ID	Serovar											
	Har	Sej	Aus	Bal	Bra	Aut	Gri	Ict	Pom	Tar	Bat	Can
12Ue1157	1:400						1:400					
13Ue0703	1:400					1:200						
13Ue0920	1:400		1:400									
13Ue1137	1:200											
13Ue1300	1:1600	1:3200								1:100		
13Ue1475	1:3200											
13Ue1631	1:400		1:3200		1:3200							
13Ue1769	1:3200											
14A0004	1:3200											
14A0027	1:3200	1:1600										
14A0032			1:3200									
14A0035			1:3200									
14A0051	1:800											
14A0057		1:200										
14A0078	1:3200											
14A0088			1:3200		1:3200							
14A0090	1:3200											
15A0004	1:1600	1:200										
15A0019	1:800											
15A0060		1:400										
15A0063	1:1600	1:400										
15A0082	1:1600	1:400										
15A0086	1:1600			1:400								
15A0093			1:1600		1:400	1:800						
15A0103	1:400											
15A0107	1:800	1:100										
15A0112	1:1600	1:200										
15A0114	1:800	1:200										
15A0122	1:400											
15A0127	1:400											
15A0135	1:400											
15A0137	1:800								1:400			
15A0146	1:800	1:100										
15A0147				1:200								
15A0149	1:800	1:400										
15A0157	1:400	1:200										
15A0162	1:100	1:200										
15A0167	1:800											
15A0171				1:200				1:800				
Total no.	31	14	6	3	3	2	1	1	1	1	0	0

*Har* Hardjo, *Sej* Sejroe, *Aus* Australis, *Bal* Ballum, *Bra* Bratislava, *Aut* Autumnalis, *Gri* Grippotyphosa, *Ict* Icterohaemorrhagiae, *Pom* Pomona, *Tar* Tarassovi, *Bat* Bataviae, *Can* Canicola

### Detection by molecular analysis

**Fig. 1:**
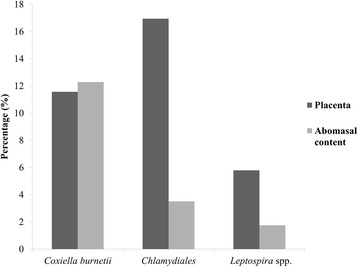
Incidence of placenta and abomasal content samples that were positive by real-time PCR for *Coxiella burnetii*, *Chlamydiales* and/or pathogenic *Leptospira* spp.

Real-time PCR detection of *C. burnetii* was positive in 28/242 (11.6%) placenta and 7/57 (12.3%) abomasal content samples. The pan-*Chlamydiales* real-time PCR was positive for 41/242 placenta (16.9%) and 2/57 (3.5%) abomasal content samples. The results after amplicon sequencing of positive samples with a Ct ≤ 35 are summarized in Table 2. *C. abortus* was detected in 24 placenta samples and in one abomasal content sample*.* New *Chlamydia*-related bacteria were detected in four placenta samples. All four were distantly related to known species but all four could be assigned to the *Parachlamydiaceae* family since they exhibited >90% similarity with at least a member of this clade. The sequencing of the remaining 12 samples was not discriminatory because of superposition of sequences, probably due to the presence of more than one member of the *Chlamydiales* order in the sample. Real-time PCR for the detection of *Leptospira* spp. was positive for 14/242 (5.8%) samples of placenta and 1/57 (1.8%) samples of abomasal contents. For seven of the samples positive by real-time PCR, serum for analysis by MAT was available and was positive in five cases (serovar Hardjo, *n* = 4; serovar Icterohaemorrhagiae, *n* = 1) and negative in two cases. All the results of the molecular analyses are included in Additional file 2: Table S2 and the percentage of positive placenta and abomasal content samples is summarized in Fig. 1.

**Table 2:** Sequence results of *Chlamydiales* samples positive by real-time PCR. Not interpretable: presence of multiple peaks

Sample ID	Organ	Related microorganism	GenBank accession no.	Similarity %
12Ue0622	Placenta	Not interpretable	–	–
12Ue1119	Placenta	*Chlamydia abortus*	Z49871	100
12Ue1503	Placenta	Uncultured *Chlamydiales* bacterium clone HE210023biof	JX083111	99.3
12Ue1510	Placenta	*Chlamydia abortus*	NR_036834	100
13Ue0490	Placenta	*Chlamydia abortus*	Z49871	94.7
13Ue0499	Placenta	Not interpretable	–	–
13Ue0815	Placenta	*Chlamydiales* bacterium cvE71	JF706724	96
13Ue0857	Placenta	*Chlamydia abortus*	Z49871	100
13Ue1009	Placenta	*Chlamydia abortus*	Z49871	99.3
13Ue1293	Placenta	*Chlamydia abortus*	Z49871	100
13Ue1359	Placenta	*Chlamydia abortus*	Z49871	100
14A0078	Placenta	*Chlamydia abortus*	Z49871	98.5
15A0068	Placenta	*Chlamydia abortus*	Z49871	90.8
15A0076	Placenta	Not interpretable	–	–
15A0078	Placenta	*Chlamydia abortus*	Z49871	99.5
15A0079	Placenta	*Chlamydia abortus*	Z49871	99.8
15A0080	Placenta	*Chlamydia abortus*	Z49871	99.5
15A0082	Placenta	*Chlamydia abortus*	Z49871	96.2
15A0087	Placenta	Uncultured *Chlamydiales* bacterium clone P-9	AF364575	97
15A0091	Placenta	*Chlamydia abortus*	Z49871	92
15A0092	Placenta	*Chlamydia abortus*	Z49871	100
15A0093	Placenta	*Chlamydia abortus*	Z49871	90.8
15A0096	Placenta	*Chlamydia abortus*	Z49871	99.5
15A0096	Abomasal content	*Chlamydia abortus*	Z49871	99.5
15A0099	Placenta	Not interpretable	–	–
15A0104	Placenta	*Chlamydia abortus*	Z49871	100
15A0107	Placenta	Not interpretable	–	–
15A0111	Placenta	*Chlamydia abortus*	Z49871	99.5
15A0113	Placenta	*Chlamydia abortus*	Z49871	100
15A0114	Placenta	*Chlamydia abortus*	Z49871	99.5
15A0117	Placenta	Not interpretable	–	–
15A0118	Placenta	*Chlamydia abortus*	Z49871	96.8
15A0121	Placenta	*Chlamydia abortus*	Z49871	98.1
15A0122	Placenta	*Chlamydia abortus*	Z49871	97.6
15A0126	Abomasal content	Not interpretable	–	–
15A0129	Placenta	Not interpretable	–	–
15A0137	Placenta	Not interpretable	–	–
15A0148	Placenta	Not interpretable	–	–
15A0155	Placenta	*Parachlamydia acanthamoebae* strain Bn9	NR_026357	90.3
15A0160	Placenta	Not interpretable	–	–
15A0172	Placenta	Not interpretable	–	–

**Fig. 2:**
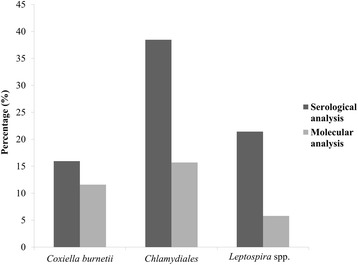
Incidence of seropositive cases and their corresponding samples that were positive by real-time PCR for *Coxiella burnetii*, *Chlamydiales* and/or pathogenic *Leptospira* spp.

To compare serological and molecular techniques we analyzed 182 cases that were processed by ELISA, MAT and PCR (Fig. 2). All three pathogens had more positive results in the serological analyses than in the molecular analysis, with *C. abortus* having the highest seropositivity. Results from samples that were positive by real-time PCR for more than one pathogen are summarized in Table 3.

**Table 3:** Results of six cases of bovine abortion which were positive for more than one pathogen

Lab ID	Cox-ELISA	Cab-ELISA	Lep MAT	Lep Serovar	Organ	mod-ZN Cox-Chl	Cox-qPCR	Chl-qPCR	Lep-qPCR
12Ue0622	NA	NA	NA		AC	–	+	–	–
					PL	–	+	+	–
13Ue1009	+	S	–		PL	–	+	+	–
14A0078	–	–	+	Har	PL	–	+	+	–
15A0087	–	S	–		PL	–	+	+	–
15A0092	–	S	–		PL	–	–	+	+
15A0099	–	–	–		PL	–	+	+	–
15A0107	+	–	+	Har/Ser	PL	–	+	+	+

Cox *Coxiella burnetii*, Cab *Chlamydia abortus*, *Lep* pathogenic *Leptospira* spp., *MAT* Microscopic Agglutination Test, *PL* placenta, *AC* abomasal content, *mod-ZN* Stamp’s modification of the Ziehl-Neelsen stain, *Chl Chlamydiales*, *NA* not available, **+**: positive result, **−**: negative result, *S* suspect positive, *Har* Hardjo, *Sej* Sejroe, *Aus* Australis, *Bal* Ballum, *Bra* Bratislava, *Aut* Autumnalis, *Gri* Grippotyphosa, *Ict* Icterohaemorrhagiae, *Pom* Pomona, *Tar* Tarassovi. The serovars are in descending order regarding the titer

### Histopathology and IHC

The severity of the placentitis, necrosis, inflammatory cell infiltrate and vasculitis in all real-time PCR-positive cases of *C. burnetii*, *Chlamydiales* and *Leptospira* spp. that were evaluated histologically (when tissue was available and not autolytic) varied greatly and the histological findings are summarized in Table 4.

**Table 4:** Histological lesions in placentas from *Coxiella burnetii*, *Chlamydiales* and *Leptospira* spp. positive cases by real-time PCR

Sample ID	Placentitis	Necrosis	Type of infiltrate	Vasculitis	Presence of ICB^a^	Presence of ECB^b^	IHC
pos *C. burnetii* (*n* = 13)							
12Ue0622	Moderate	Moderate	Mixed	Yes	Yes	No	Neg
13Ue0536	Moderate	Mild	Mixed	No	No	No	Neg
13Ue0858	Mild	Moderate	Mixed	No	Yes	No	Neg
13Ue1008	Moderate	Moderate	Mixed	Yes	No	No	Neg
13Ue1009	Moderate	Moderate	Mixed	Yes	No	No	Neg
13Ue1414	Mild	Mild	Neutrophilic	No	Yes	Yes	Neg
13Ue1488	Mild	Moderate	Neutrophilic	No	Yes	Yes	Neg
13Ue1524	Mild	Mild	Mixed	Yes	Yes	Yes	Neg
13Ue1644	No	No	No	No	No	No	Neg
14A0076	Mild	No	Neutrophilic	No	No	Yes	Neg
15A0086	Mild	Moderate	Mixed	No	Yes	Yes	Neg
15A0101	Moderate	Moderate	Mixed	No	No	Yes	Neg
15A0107	Severe	Severe	Mixed	Yes	Yes	No	Neg
pos *Chlamydiales* (*n* = 14)							
12Ue0622	Moderate	Moderate	Mixed	Yes	Yes	No	Neg
12Ue1503	Mild	Mild	Mixed	No	No	Yes	Neg
13Ue1009	Moderate	Moderate	Mixed	Yes	No	No	Neg
15A0076	Moderate	Moderate	Mixed	Yes	Yes	Yes	Neg
15A0078	Severe	Moderate	Mixed	No	No	Yes	Neg
15A0080	Moderate	Moderate	Mixed	No	No	Yes	Neg
15A0082	Severe	Mild	Mixed	No	No	Yes	Neg
15A0093	Mild	Moderate	Mixed	No	No	Yes	Neg
15A0104	Moderate	Mild	Mixed	Yes	No	Yes	Neg
15A0107	Severe	Severe	Mixed	Yes	Yes	No	Neg
15A0121	Mild	Mild	Mononuclear	No	No	Yes	Neg
15A0122	Mild	Moderate	Neutrophilic	No	No	Yes	Neg
15A0137	Severe	Moderate	Mixed	Yes	Yes	Yes	Neg
15A0148	Moderate	Moderate	Mixed	Yes	No	Yes	Neg
pos *Leptospira* spp. (*n* = 5)							
12Ue1016	Severe	Mild	Mixed	No	No	Yes	Neg
12Ue1185	Severe	Mild	Mixed	No	Yes	Yes	Neg
15A0011	Mild	Mild	Mononuclear	No	No	Yes	Neg
15A0107	Severe	Severe	Mixed	Yes	Yes	No	Neg
15A0127	Mild	Mild	Mononuclear	No	No	Yes	Neg

^a^Presence of intracytoplasmic bacteria (ICB). ^b^Presence of extracellular bacteria (ECB)

Regardless of the etiological agent, if necrosis was present in the cotyledon it was multifocal, randomly distributed and affected the chorioallantoic stroma and the villi including the trophoblasts.

**Fig. 3:**
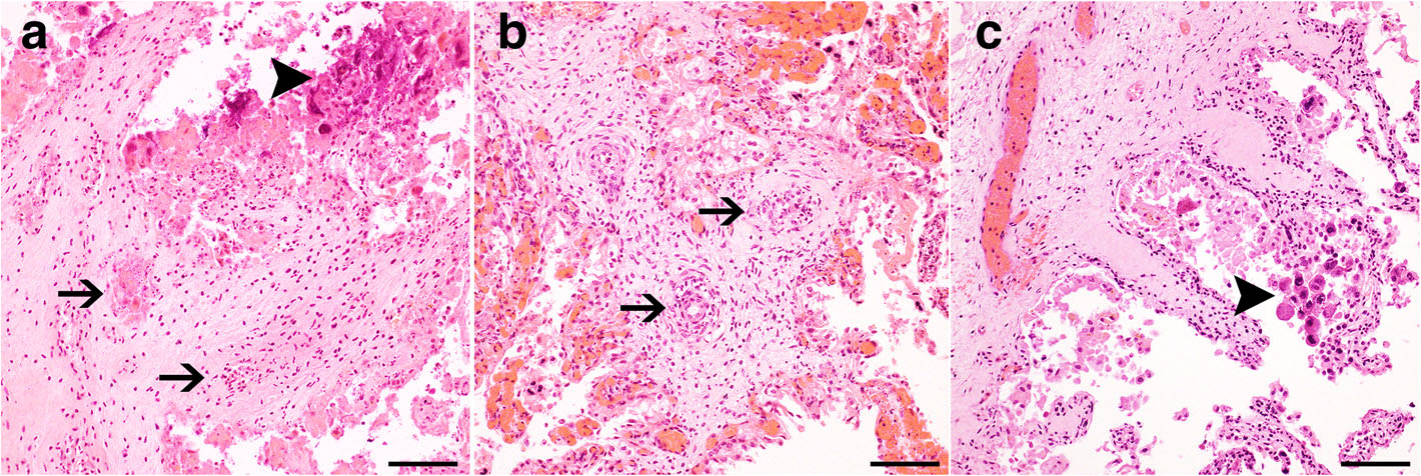
Histopathology of representative placental samples from bovine abortions positive by real-time PCR for: *Coxiella burnetii* (**a**), *Chlamydia abortus* (**b**) and pathogenic *Leptospira* spp. (**c**). Infection with either *C. burnetii* (**a**) or *C. abortus* (**b**) was characterized by variable degrees of vasculitis (arrow) and necrosis (arrowhead). Cases positive for pathogenic *Leptospira* spp. by real-time PCR (**c**) showed variable severities of necrosis (arrowhead) and lacked vasculitis. HE, bar 100 μm

Placentitis was present in 12 of 13 samples that were positive for *C. burnetii* by real-time PCR. Necrosis was present in 11 of 13 cases. Mixed inflammatory infiltrates characterized by neutrophils, macrophages and lymphocytes were present in nine of 13 cases and only three cases were designated as suppurative placentitis. Vasculitis was present in five of 13 cases and characterized by infiltration of neutrophils, macrophages and lymphocytes primarily in the tunicae media and adventitia resulting in mild fibrinoid necrosis only (Fig. 3a). All cases positive for *Chlamydiales* by real-time PCR had placentitis and necrosis. Mixed inflammatory cell infiltrates were present in 12 of 14 cases, only one was designated suppurative and one case was infiltrated by macrophages and lymphocytes (mononuclear) only. Vasculitis was present in seven of the 14 cases (Fig. 3b). Similarly, all cases of *Leptospira* spp. positive by real-time PCR had placentitis and necrosis (Fig. 3c). Mixed inflammatory cell infiltrates were present in three of five cases, while in two cases the inflammation was comprised of mononuclear leukocytes only. For cases positive for *Leptospira* spp. by real-time PCR no suppurative placentitis was found and only one case had vasculitis.

Immunohistochemical analysis failed to visualize *C. burnetii*, *Chlamydiales* and *Leptospira* spp. in all sections evaluated (Table 4).

### Statistical analysis

The Cohen’s kappa coefficient is summarized in Table 5. The degree of agreement between the serological and the molecular diagnostic techniques for *C. burnetii*, *C. abortus* and *Leptospira* spp. was poor with κ = 0.103 ± 0.086, κ = −0.006 ± 0.067 and κ = 0.163 ± 0.074, respectively.

**Table 5:** Cohen’s kappa (κ) coefficient with 95% of CIs to assess the degree of agreement between molecular and serological techniques for the diagnosis of *C. burnetii*, *C. abortus* and *Leptospira* spp.

	*C. burnetii*	*C. abortus*	*Leptospira* spp.
Number of observed agreements	145 (79.67% of the observations)	103 (56.59% of the observations)	146 (80.22% of the observations)
Number of agreements expected by chance	140.7 (77.33% of the observations)	103.5 (56.85% of the observations)	139.0 (6.37% of the observations)
Kappa (κ)	0.103 ± 0.086	−0.006 ± 0.067	0.163 ± 0.074
95% CIs	−0.065 to 0.272	−0.137 to 0.126	0.018 to 0.308
Strength of agreement	Poor	Poor	Poor

## Discussion

This study investigated, concomitantly, the prevalence of *C. burnetii*, *C. abortus* and pathogenic *Leptospira* spp. DNA in bovine abortion material and seroconversion in affected dams and highlights the underestimation associated with using a single staining technique. Although detection of any of these pathogens does not equate invariably to causality with respect to bovine abortion, their presence does invariably represent a high zoonotic risk and a possible reservoir of infection for other animals.

The frequency of antibodies specific for *C. burnetii* in dams was 15.9%, comparable to the reported seroprevalence of 16.7% in aborting cows in Switzerland by Hässig and Lubsen [40]. However, previous studies reported similar seropositivity for *C. burnetii* antibodies but in healthy cows and in different European countries (Bulgaria 20.8%; France 15%; Germany 19.3% and the Netherlands 21%) [41] suggesting serological results are not reliable for diagnostic purposes. With 38.4% positive and 13.0% suspect positive reactions the seropositivity to anti-*C. abortus* antibodies was the highest of the three abortifacient pathogens investigated in this study. This result was in agreement with studies in other countries which also reported a high prevalence of anti-chlamydial antibodies in cattle, with seropositivity ranging from 45% to 100% [42]. However, such high seropositivity rates have to be interpreted with caution. Firstly, a single seropositive result is not necessarily related to the etiology of the abortion and might be due to a previous exposure [43]. Secondly, serological tests may not be *C. abortus* specific and positive titers can arise from cross-reactivity to *C. pecorum*, a common intestinal opportunistic chlamydial species found in ruminants [44]. The frequency of *Leptospira* spp. antibodies was 21.4% and primarily due to serovar Hardjo (seroprevalence of 17.0%). The implication of a positive titer to serovar Hardjo on fetal loss remains controversial as many studies failed to show a causal association between seropositivity and abortion [22, 45–48], while others described Hardjo as a cause of abortions [49, 50]. The interpretation of the serological results for *Leptospira* spp. should be done carefully since there is cross-reactivity between serovars of the same serogroup; nevertheless, the infecting serovar is more likely to show the higher titer [22, 51]. Although Sejroe was the second most prevalent serovar (14 cases), 12 cases were positive for serovars Hardjo and Sejroe, belonging to the same serogroup. Yet, Hardjo presented the higher titer in 10 cases making Australis (six cases) the second most frequent serovar. Abortion in cattle due to serovar Hardjo is a chronic event with a variable serological response at the time of abortion [22] and confirmation of infection by MAT is difficult because maternal antibody production mostly occurs prior to fetal death [52].

Molecular detection of DNA of abortifacient agents has been shown to be highly sensitive and specific [37, 53–55]. By real-time PCR we detected *C. burnetii* in 12.1% of cases, similar to findings in Italy (11.3%) obtained by nested PCR [56] but lower than results by classical PCR from Portugal (17.2%) [57] and by real-time PCR from Hungary (25.9%) [58]. These findings, although obtained with different techniques, may reflect different endemicity. Furthermore, we showed the capacity of *C. burnetii* of spreading via the amniotic-oral route [59, 60] with the 7/57 samples of abomasal content being positive. Of the 21 real-time PCR positive cases of *C. burnetii*, 15 were seronegative suggesting early stages of infection when antibodies are not yet present, or environmental contamination of samples or failure of the dam to seroconvert occurred. In contrast, 23 cases with positive sera were negative by real-time PCR suggesting previous exposure to *C. burnetii* is not uncommon. The statistical analysis showed a poor agreement (κ = 0.103 ± 0.086) between the serological and the molecular technique indicating that there is a poor relationship between the seropositivity of the dam for antibodies to *C. burnetii* and an abortion event as reported previously [61–64]. It is important to keep in mind that real-time PCR is highly sensitive and thus able to detect low levels of *C. burnetii*. Yet, different strains harbor a very variable number of the target IS*1111* (between 7 and 110) making quantification inaccurate for this bacterium [65]. For the final interpretation at herd-level it is recommended to include complementary techniques and consider the case history [18, 66].

Of 43 real-time PCR-positive samples for *Chlamydiales* (placenta, *n* = 41; abomasal content, *n* = 2) *C. abortus* could be identified by sequencing in 9.6% of the cases, although the prevalence could be higher because in 12 samples a single species could not be assigned due to multiple peaks. In Eastern Switzerland, *C. abortus* was considered not to play an important role in bovine abortion in studies by end-point PCR [39] and real-time PCR [67]. However, Blumer et al. [9] confirmed the presence of *C. abortus* in 14.8% of studied cases of abortion from Eastern Switzerland. We could detect members of the *Parachlamydiaceae* family in four samples confirming that *Chlamydia*-related bacteria could be involved in bovine abortion as reported previously [9, 39, 67] and could cause mixed infection [68]. It is noteworthy that some samples with *Chlamydia*-related bacteria, including *P. acanthamoebae*, were positive by *C. abortus* ELISA also. This result might be due to the production of antibodies that could cross-react with other chlamydial-species due to a genus-specific epitope of the lipopolysaccharide [44, 69–71]. This might also be the underlying reason for the poor agreement (κ = −0.006 ± 0.067) between the serological and the molecular technique.

In six cases of coxiellosis we found evidence of coinfection with *C. abortus* and *Chlamydiales*-related bacteria. Although *C. burnetii* and *Chlamydiales* belong to phylogenetically unrelated species [72], they have some similarities in their interaction with the host and mechanisms of pathogenicity [73]. Thus, the diagnosis of either agent is usually established by microscopic examination of stained placenta smears in veterinary diagnostic laboratories but this cannot discriminate between the different organisms. Pritchard et al. [74] stated that the mod-ZN stain is insufficiently sensitive in cattle cotyledons. Our findings agree with this and confirm that the mod-ZN stain is not very sensitive for the detection of either *C. burnetii* or *Chlamydiales* infection in bovine abortion material and that it should be replaced by specific real-time PCRs.

Pathogenic *Leptospira* spp. had a prevalence of 5.6% by real-time PCR (placenta, *n* = 14; abomasal content, *n* = 1). The detection of leptospires in internal organs of aborted or stillborn fetuses reflects chronic leptospirosis of the mother and indicates an active infection of the fetus, but PCR-based diagnosis of leptospirosis alone cannot identify the infecting serovar; moreover, contamination with faeces or autolysis in clinical samples is known to lead to false-negative results [22]. Hence, the combination of both, molecular and serological techniques is of epidemiological value, even though no satisfactory agreement between techniques (κ = 0.163 ± 0.074) was achieved. Unfortunately, in only seven cases material was available for both analysis, and four sera of these were positive for serovar Hardjo and one for serovar Icterohaemorrhagiae. Two of the samples positive by real-time PCR for *Leptospira* spp. DNA were negative in MAT indicating an early stage of the infection or failure to detect seroconversion. In one case, pathogenic *Leptospira* spp., presumably identified as serovar Hardjo by serology, were detected together with *C. burnetii* and *Chlamydiales* and in another case we found possible coinfection between pathogenic *Leptospira* spp. and *C. abortus*.

Histological investigation and confirmation of the cellular inflammatory process indicative of infectious agents is important to unambiguously confirm the implication of a specific etiological agent especially if it could also be present in the commensal and the environmental microbiota [43]. However, as the cotyledonary lesions are not pathognomonic for any of the three pathogens investigated [39, 75], a definitive diagnosis based on histopathology only is not possible. Accordingly, in real-time PCR-positive cases of *C. burnetii* and *Chlamydiales* we found similar placental lesions varying only in the degree and severity of the inflammatory infiltrate*.* Although, vasculitis in the placenta of abortion cases is described as a prominent feature of *C. abortus* infections [39], it is not invariably present. Furthermore, vasculitis in the placenta is present frequently in cases of *C. burnetii* abortion [75, 76] as was observed in this study. Additionally, we found that not all *Chlamydiales* real-time PCR-positive cases displayed vasculitis, similar to previous reports [9, 39]. All *Leptospira* spp. real-time PCR-positive cases showed necrotizing placentitis with three and two displaying mixed and mononuclear inflammatory infiltrates, respectively. Vasculitis was not observed in any sample except one case which was real-time PCR-positive for *C. burnetii* and *Chlamydiales* also. Placental lesions caused by *Leptospira* spp. in bovine abortion are not well characterized but, based on our limited observations, vasculitis is not a prominent feature.

Lesion-associated pathogen detection is usually considered vital for definitive diagnosis to prove causality. However, we were not able to identify lesion-associated *C. burnetii*, *Chlamydiales* or *Leptospira* spp. by IHC in any of the analyzed slides. IHC is known to have lower sensitivity than real-time PCR [77, 78], especially when there is some degree of autolysis in the samples as is often the case for abortion material.

Limited first-line diagnostics (mod-ZN staining) could only detect possible abortifacient agents in 11 cases (4.4%) while real-time PCR detected a possible abortifacient agent in 78 cases (31.2%). The fact that (i) *C. burnetii, Chlamydiales* and *Leptospira* spp. are all difficult to culture, (ii) serology cannot exclude a past infection or confirm an ongoing infection and (iii) IHC apparently fails to demonstrate the presence of the agents, makes the molecular approach the method of choice.

## Conclusions

In conclusion, we recommend an extended workflow including molecular analysis for routine abortion diagnostics to avoid the underestimation of the discussed agents and histological analysis to avoid misinterpretation of real-time PCR positive results. It would be prudent to use molecular methods initially and then subject positive cases to histological screening. For further epidemiological investigations complementary serological analyses should be considered. However, the real value of this work was determining the inherent public health risk with respect to these zoonotic pathogens and their prevalence in bovine abortion material as important source of infection.

## Additional files

Additional file 1: Table S1. The 12 *Leptospira* spp. strains used as live antigens in the Microscopic Agglutination test (MAT) obtained from the Royal Tropical Institute (KIT), Amsterdam (The Netherlands). (DOCX 14 kb)

Additional file 2: Table S2. Results of the 249 cases of bovine abortion analyzed in this study. Cox: *Coxiella burnetii*; Cab: *Chlamydia abortus*; Lep; pathogenic *Leptospira* spp.; MAT: Microscopic Agglutination Test; PL: placenta; AC: Abomasal content; mod-ZN: Stamp’s modification of the Ziehl-Neelsen stain; Chl: *Chlamydiales*; Ct: threshold cycle value; NA: not available, **+**: positive result, **−**: negative result, S: suspect positive, Har: Hardjo, Sej: Sejroe, Aus: Australis, Bal: Ballum, Bra: Bratislava, Aut: Autumnalis, Gri: Grippotyphosa, Ict: Icterohaemorrhagiae, Pom: Pomona, Tar: Tarassovi. The serovars are in descending order regarding the titer. (DOCX 126 kb)
