# Supplementary material for: Neglected zoonotic agents in cattle abortion: tackling the difficult to grow bacteria
Source: BMC Vet Res. 2017 Dec 2;13:373. doi: 10.1186/s12917-017-1294-y (PMC5712085; doi:10.1186/s12917-017-1294-y)
Supplement: Supplementary file 1 — The 12 Leptospira spp. strains used as live antigens in the Microscopic Agglutination test (MAT) obtained from the Royal Tropical Institute (KIT), Amsterdam (The Netherlands). (DOCX 14 kb) [file 12917_2017_1294_MOESM1_ESM.docx]

| **Table S1.** The 12 *Leptospira* spp. strains used as live antigens in the Microscopic Agglutination test (MAT) obtained from the Royal Tropical Institute (KIT), Amsterdam (The Netherlands). | | | |
| --- | --- | --- | --- |
| **Genomospecies** | **Serogroup** | **Serovar** | **Strain** |
| *L.interrogans* | Australis | Australis | Ballico |
|  | Australis | Bratislava | Jez-Bratislava |
|  | Autumnalis | Autumnalis | Akiyami |
|  | Bataviae | Bataviae | Swart |
|  | Canicola | Canicola | Hond Utrecht IV |
|  | Icterohaemorrhagiae | Icterohaemorrhagiae | RGA |
|  | Pomona | Pomona | Pomona |
|  | Sejroe | Hardjo | Hardjoprajitno |
| *L.borgpetersenii* | Ballum | Ballum | Mus127 |
|  | Tarassovi | Tarassovi | Perepelitsin |
|  | Sejroe | Sejroe | M84 |
| *L.kirschneri* | Grippotyphosa | Grippotyphosa | Moskva V |
