# Supplementary material for: Neglected zoonotic agents in cattle abortion: tackling the difficult to grow bacteria
Source: BMC Vet Res. 2017 Dec 2;13:373. doi: 10.1186/s12917-017-1294-y (PMC5712085; doi:10.1186/s12917-017-1294-y)
Supplement: Supplementary file 2 — Results of the 249 cases of bovine abortion analyzed in this study. Cox: Coxiella burnetii; Cab: Chlamydia abortus; Lep; pathogenic Leptospira spp.; MAT: Microscopic Agglutination Test; PL: placenta; AC: Abomasal content; mod-ZN: Stamp’s modification of the Ziehl-Neelsen stain; Chl: Chlamydiales; Ct: threshold cycle value; NA: not available, +: positive result, −: negative result, S: suspect positive, Har: Hardjo, Sej: Sejroe, Aus: Australis, Bal: Ballum, Bra: Bratislava, Aut: Autumnalis, Gri: Grippotyphosa, Ict: Icterohaemorrhagiae, Pom: Pomona, Tar: Tarassovi. The serovars are in descending order regarding the titer. (DOCX 126 kb) [file 12917_2017_1294_MOESM2_ESM.docx]

| **Table S2.** Results of the 249 cases of bovine abortion analyzed in this study. Cox: *Coxiella burnetii*; Cab: *Chlamydia abortus*; Lep; pathogenic *Leptospira* spp.; MAT: Microscopic Agglutination Test; PL: placenta; AC: Abomasal content; mod-ZN: Stamp’s modification of the Ziehl-Neelsen stain; Chl: *Chlamydiales*; Ct: threshold cycle value; NA: not available, **+**: positive result, **-**: negative result, S: suspect positive, Har: Hardjo, Sej: Sejroe, Aus: Australis, Bal: Ballum, Bra: Bratislava, Aut: Autumnalis, Gri: Grippotyphosa, Ict: Icterohaemorrhagiae, Pom: Pomona, Tar: Tarassovi. The serovars are in descending order regarding the titer. | | | | | | | | | |
| --- | --- | --- | --- | --- | --- | --- | --- | --- | --- |
| Lab ID | Cox-ELISA | Cab-ELISA | Lep  MAT | Lep  Serovar | Organ | mod-ZN Cox-Chl | Cox-qPCR (Ct) | Chl-qPCR (Ct) | Lep-qPCR (Ct) |
| 12Ue0602 | NA | NA | NA |  | PL | - | + (17.80) | - | - |
| 12Ue0622 | NA | NA | NA |  | AC | - | + (31.17) | - | - |
|  |  |  |  |  | PL | - | + (33.21) | + (30.72) | - |
| 12Ue0638 | NA | NA | NA |  | PL | - | - | - | - |
| 12Ue0784 | NA | NA | NA |  | PL | + | + (15.83) | - | - |
| 12Ue0815 | NA | NA | NA |  | PL | + | + (16.99) | - | - |
| 12Ue0876 | NA | NA | NA |  | PL | - | - | - | + (38.76) |
| 12Ue0890 | NA | NA | NA |  | PL | - | - | - | + (37.03) |
| 12Ue0928 | NA | NA | NA |  | AC | - | - | - | - |
|  |  |  |  |  | PL | - | - | - | - |
| 12Ue1015 | NA | NA | NA |  | AC | - | - | - | - |
|  |  |  |  |  | PL | - | - | - | - |
| 12Ue1016 | NA | NA | NA |  | AC | - | - | - | - |
|  |  |  |  |  | PL | - | - | - | + (39.88) |
| 12Ue1034 | NA | NA | NA |  | PL | - | - | - | - |
| 12Ue1049 | NA | NA | NA |  | PL | - | - | - | - |
| 12Ue1050 | NA | NA | NA |  | PL | - | - | - | - |
| 12Ue1052 | NA | NA | NA |  | PL | - | - | - | - |
| 12Ue1059 | NA | NA | NA |  | PL | - | - | - | - |
| 12Ue1062 | NA | NA | NA |  | PL | - | - | - | + (36.08) |
| 12Ue1074 | NA | NA | NA |  | PL | - | + (36.98) | - | - |
| 12Ue1086 | NA | NA | NA |  | PL | - | - | - | - |
| 12Ue1095 | NA | NA | NA |  | PL | - | - | - | - |
| 12Ue1096 | NA | NA | NA |  | AC | - | - | - | - |
|  |  |  |  |  | PL | - | - | - | - |
| 12Ue1106 | NA | NA | NA |  | PL | - | - | - | - |
| 12Ue1110 | NA | NA | NA |  | PL | - | - | - | - |
| 12Ue1119 | NA | NA | NA |  | PL | - | - | + (30.22) | - |
| 12Ue1143 | NA | NA | NA |  | PL | - | - | - | - |
| 12Ue1147 | NA | NA | NA |  | PL | - | - | - | - |
| 12Ue1150 | NA | NA | NA |  | PL | - | - | - | - |
| 12Ue1157 | S | **-** | **+** | Har/Gri | PL | - | - | - | + (31.40) |
| 12Ue1181 | NA | NA | NA |  | AC | - | - | - | - |
|  |  |  |  |  | PL | - | - | - | - |
| 12Ue1185 | NA | NA | NA |  | AC | - | - | - | + (37.25) |
|  |  |  |  |  | PL | - | - | - | + (36.42) |
| 12Ue1228 | NA | NA | NA |  | AC | - | - | - | - |
|  |  |  |  |  | PL | - | - | - | - |
| 12Ue1278 | NA | NA | NA |  | AC | - | - | - | - |
|  |  |  |  |  | PL | - | - | - | - |

**Table S2 – *continued***

| Lab ID | Cox-ELISA | Cab-ELISA | Lep  MAT | Lep  Serovar | Organ | mod-ZN Cox-Chl | Cox-qPCR | Chl-qPCR | Lep-qPCR |
| --- | --- | --- | --- | --- | --- | --- | --- | --- | --- |
| 12Ue1390 | NA | NA | NA |  | PL | - | - | - | - |
| 12Ue1391 | NA | NA | NA |  | PL | - | - | - | - |
| 12Ue1392 | NA | NA | NA |  | PL | - | - | - | - |
| 12Ue1393 | NA | NA | NA |  | PL | - | - | - | - |
| 12Ue1410 | NA | NA | NA |  | AC | - | - | - | - |
| 12Ue1432 | NA | NA | NA |  | PL | - | - | - | + (35.91) |
| 12Ue1489 | NA | NA | NA |  | PL | - | - | - | - |
| 12Ue1503 | NA | NA | NA |  | AC | - | - | - | - |
|  |  |  |  |  | PL | - | - | + (35.47) | - |
| 12Ue1510 | NA | NA | NA |  | PL | + | - | + (20.69) | - |
| 12Ue1535 | NA | NA | NA |  | PL | - | + (14.76) | - | - |
| 12Ue1536 | NA | NA | NA |  | AC | - | - | - | - |
| 13Ue0003 | NA | NA | NA |  | PL | - | - | - | - |
| 13Ue0007 | NA | NA | NA |  | PL | - | - | - | - |
| 13Ue0052 | NA | NA | NA |  | AC | + | + (22.51) | - | - |
| 13Ue0064 | + | + | - |  | PL | - | - | - | - |
| 13Ue0066 | NA | NA | NA |  | PL | - | - | - | - |
| 13Ue0142 | NA | NA | NA |  | PL | - | - | - | - |
| 13Ue0182 | NA | NA | NA |  | PL | - | - | - | - |
| 13Ue0195 | NA | NA | NA |  | PL | - | - | - | - |
| 13Ue0203 | NA | NA | NA |  | PL | - | - | - | - |
| 13Ue0219 | NA | NA | NA |  | AC | - | - | - | - |
|  |  |  |  |  | PL | - | - | - | - |
| 13Ue0233 | NA | NA | NA |  | AC | - | - | - | - |
|  |  |  |  |  | PL | - | - | - | - |
| 13Ue0236 | NA | NA | NA |  | AC | - | - | - | - |
|  |  |  |  |  | PL | - | - | - | - |
| 13Ue0238 | NA | NA | NA |  | PL | - | - | - | - |
| 13Ue0249 | NA | NA | NA |  | PL | - | - | - | - |
| 13Ue0253 | NA | NA | NA |  | AC | - | - | - | - |
|  |  |  |  |  | PL | - | - | - | - |
| 13Ue0255 | NA | NA | NA |  | AC | - | - | - | - |
| 13Ue0268 | NA | NA | NA |  | AC | - | - | - | - |
|  |  |  |  |  | PL | - | - | - | - |
| 13Ue0274 | NA | NA | NA |  | AC | - | - | - | - |
| 13Ue0288 | NA | NA | NA |  | PL | - | - | - | - |
| 13Ue0426 | NA | NA | NA |  | PL | - | - | - | - |
| 13Ue0457 | + | + | - |  | AC | - | - | - | - |
|  |  |  |  |  | PL | - | - | - | - |
| 13Ue0475 | NA | NA | NA |  | PL | - | - | - | - |
| 13Ue0490 | NA | NA | NA |  | PL | - | - | + (31.50) | - |
| 13Ue0499 | + | - | - |  | PL | - | - | + (37.04) | - |
| 13Ue0534 | NA | NA | NA |  | PL | - | - | - | - |
| 13Ue0536 | NA | NA | NA |  | AC | - | - | - | - |
|  |  |  |  |  | PL | - | + (35.14) | - | - |

**Table S2 – *continued***

| Lab ID | Cox-ELISA | Cab-ELISA | Lep  MAT | Lep  Serovar | Organ | mod-ZN Cox-Chl | Cox-qPCR | Chl-qPCR | Lep-qPCR |
| --- | --- | --- | --- | --- | --- | --- | --- | --- | --- |
| 13Ue0703 | + | S | + | Har/Aut | PL | - | - | - | - |
| 13Ue0734 | NA | NA | NA |  | PL | - | + (25.8) | - | - |
| 13Ue0815 | + | + | - |  | PL | - | - | + (36.17) | - |
| 13Ue0839 | + | - | - |  | PL | + | + (19.08) | - | - |
| 13Ue0857 | + | + | - |  | PL | - | - | + (33.41) | - |
| 13Ue0858 | + | - | - |  | AC | - | + (28.66) | - | - |
|  |  |  |  |  | PL | + | + (17.31) | - | - |
| 13Ue0920 | + | - | + | Aus/Har | AC | - | - | - | - |
|  |  |  |  |  | PL | - | - | - | - |
| 13Ue1008 | + | S | - |  | AC | - | - | - | - |
|  |  |  |  |  | PL | - | + (37.37) | - | - |
| 13Ue1009 | + | S | - |  | PL | - | + (30.73) | + (34.15) | - |
| 13Ue1042 | + | - | - |  | PL | - | - | + (37.94) | - |
| 13Ue1080 | NA | NA | NA |  | PL | - | - | - | + (36.91) |
| 13Ue1081 | NA | NA | NA |  | PL | - | - | - | - |
| 13Ue1137 | + | - | + | Har | AC | - | - | - | - |
|  |  |  |  |  | PL | - |  | - | - |
| 13Ue1177 | - | - | - |  | PL | - | - | - | - |
| 13Ue1180 | - | + | - |  | AC | - | - | - | - |
|  |  |  |  |  | PL | - | - | - | - |
| 13Ue1181 | - | + | - |  | PL | - | - | - | - |
| 13Ue1211 | - | - | - |  | PL | - | - | - | - |
| 13Ue1271 | - | + | - |  | PL | - | - | - | - |
| 13Ue1275 | - | - | - |  | AC | - | - | - | - |
|  |  |  |  |  | PL | - | - | - | - |
| 13Ue1293 | - | + | - |  | PL | - | - | + (31.75) | - |
| 13Ue1300 | + | - | + | Ser/Har/Tar | PL | - | - | - | - |
| 13Ue1347 | - | - | - |  | PL | - | - | - | - |
| 13Ue1355 | - | - | - |  | PL | - | - | - | - |
| 13Ue1359 | - | + | - |  | PL | - | - | + (31.69) | - |
| 13Ue1360 | + | S | - |  | AC | - | - | - | - |
|  |  |  |  |  | PL | - | - | - | - |
| 13Ue1361 | - | - | - |  | PL | - | - | - | - |
| 13Ue1409 | - | - | - |  | PL | - | - | - | - |
| 13Ue1414 | - | S | - |  | AC | - | - | - | - |
|  |  |  |  |  | PL | - | + (34.71) | - | - |
| 13Ue1419 | - | + | - |  | PL | - | - | - | - |
| 13Ue1440 | - | - | - |  | PL | - | - | - | - |
| 13Ue1450 | - | - | - |  | AC | - | - | - | - |
|  |  |  |  |  | PL | - | - | - | - |
| 13Ue1475 | - | + | + | Har | PL | - | - | - | - |
| 13Ue1476 | - | - | - |  | PL | - | - | - | - |
| 13Ue1488 | - | + | - |  | AC | - | + (34.41) | - | - |
|  |  |  |  |  | PL | - | + (38.19) | - | - |
| 13Ue1524 | - | - | - |  | AC | - | + (32.57) | - | - |
|  |  |  |  |  | PL | - | + (32.12) | - | - |

**Table S2 – *continued***

| Lab ID | Cox-ELISA | Cab-ELISA | Lep  MAT | Lep  Serovar | Organ | mod-ZN Cox-Chl | Cox-qPCR | Chl-qPCR | Lep-qPCR |
| --- | --- | --- | --- | --- | --- | --- | --- | --- | --- |
| 13Ue1546 | - | - | - |  | PL | - | - | - | - |
| 13Ue1630 | - | + | - |  | PL | - | - | - | - |
| 13Ue1631 | - | + | + | Aus/Bra/Har | AC | - | - | - | - |
|  |  |  |  |  | PL | - | - | - | - |
| 13Ue1644 | - | - | - |  | AC | - | + (35.20) | - | - |
|  |  |  |  |  | PL | - | + (34.77) | - | - |
| 13Ue1650 | - | - | - |  | PL | - | - | - | - |
| 13Ue1672 | + | S | - |  | PL | - | - | - | - |
| 13Ue1696 | - | - | - |  | PL | - | - | - | - |
| 13Ue1697 | S | + | - |  | PL | - | - | - | - |
| 13Ue1714 | + | + | - |  | AC | - | - | - | - |
|  |  |  |  |  | PL | - | - | - | - |
| 13Ue1756 | - | S | - |  | AC | - | - | - | - |
|  |  |  |  |  | PL | - | - | - | - |
| 13Ue1769 | - | S | + | Har | PL | - | - | - | + (39.32) |
| 13Ue1797 | - | S | - |  | PL | - | + (35.70) | - | - |
| 13Ue1806 | - | + | - |  | PL | - | - | - | - |
| 13Ue1832 | - | - | - |  | PL | - | - | - | - |
| 13Ue1836 | - | + | - |  | PL | - | - | - | - |
| 14A0002 | - | - | - |  | PL | - | - | - | - |
| 14A0003 | - | - | - |  | PL | - | - | - | - |
| 14A0004 | - | - | + | Har | PL | - | - | - | - |
| 14A0005 | - | + | - |  | PL | - | - | - | - |
| 14A0012 | - | + | - |  | PL | - | - | - | - |
| 14A0013 | - | + | - |  | PL | - | - | - | - |
| 14A0015 | - | S | - |  | AC | - | - | - | - |
| 14A0020 | - | S | - |  | AC | - | - | - | - |
|  |  |  |  |  | PL | - | - | - | - |
| 14A0023 | - | + | - |  | AC | - | - | - | - |
|  |  |  |  |  | PL | - | - | - | - |
| 14A0026 | - | - | - |  | AC | - | - | - | - |
|  |  |  |  |  | PL | - | - | - | - |
| 14A0027 | - | + | + | Har/Ser | PL | - | - | - | - |
| 14A0032 | + | - | + | Aus | PL | + | - | + (38.02) | - |
| 14A0034 | - | + | - |  | PL | - | + (31.74) | - | - |
| 14A0035 | + | + | + | Aus | PL | - | - | - | - |
| 14A0036 | - | - | - |  | PL | - | - | - | - |
| 14A0037 | - | - | - |  | PL | - | - | - | - |
| 14A0044 | - | - | - |  | PL | - | - | - | - |
| 14A0045 | + | S | - |  | PL | + | + (33.77) | - | - |
| 14A0046 | - | S | - |  | AC | - | - | - | - |
|  |  |  |  |  | PL | - | - | - | - |
| 14A0048 | - | + | - |  | PL | - | - | - | - |

**Table S2 – *continued***

| Lab ID | Cox-ELISA | Cab-ELISA | Lep  MAT | Lep  Serovar | Organ | mod-ZN Cox-Chl | Cox-qPCR | Chl-qPCR | Lep-qPCR |
| --- | --- | --- | --- | --- | --- | --- | --- | --- | --- |
| 14A0051 | - | - | + | Har | AC | - | - | - | - |
|  |  |  |  |  | PL | - | - | - | - |
| 14A0052 | - | - | - |  | PL | - | + (36.15) | - | - |
| 14A0053 | - | - | - |  | PL | - | - | - | - |
| 14A0054 | - | - | - |  | AC | - | - | - | - |
|  |  |  |  |  | PL | - | - | - | - |
| 14A0056 | - | + | - |  | AC | - | - | - | - |
|  |  |  | - |  | PL | - | - | - | - |
| 14A0057 | - | - | + | Ser | AC | - | - | - | - |
|  |  |  |  |  | PL | - | - | - | - |
| 14A0076 | - | + | - |  | AC | - | - | - | - |
|  |  |  |  |  | PL | - | + (36.11) | - | - |
| 14A0078 | - | - | + | Har | PL | - | + (34.43) | + (33.11) | - |
| 14A0079 | - | - | - |  | PL | - | - | - | - |
| 14A0082 | - | + | - |  | PL | - | - | - | - |
| 14A0083 | - | - | - |  | PL | - | - | - | - |
| 14A0084 | - | + | - |  | PL | - | - | - | - |
| 14A0088 | - | - | + | Aus/Bra | AC | - | - | - | - |
|  |  |  |  |  | PL | - | - | - | - |
| 14A0090 | - | + | + | Har | AC | - | + (37.82) | - | - |
|  |  |  |  |  | PL | - | - | - | - |
| 14A0107 | - | + | - |  | PL | - | - | - | - |
| 14A0113 | - | + | - |  | AC | - | - | - | - |
|  |  |  | - |  | PL | - | - | - | - |
| 14A0143 | - | S | - |  | AC | - | - | - | - |
| 14A0144 | - | + | - |  | AC | + | - | - | - |
|  |  |  | - |  | PL | + | - | - | - |
| 15A0004 | - | S | + | Har/Ser | AC | - | - | - | - |
|  |  |  |  |  | PL | - | - | - | - |
| 15A0006 | + | - | - |  | PL | - | - | - | - |
| 15A0008 | - | + | - |  | PL | - | - | - | - |
| 15A0009 | - | + | - |  | PL | - | - | - | - |
| 15A0011 | - | - | - |  | PL | - | - | - | + (37.37) |
| 15A0015 | - | - | - |  | PL | - | - | - | - |
| 15A0016 | - | + | - |  | PL | - | - | - | - |
| 15A0017 | - | + | - |  | PL | - | - | - | - |
| 15A0018 | - | - | - |  | PL | - | - | - | - |
| 15A0019 | - | + | + | Har | PL | - | - | - | - |
| 15A0020 | - | - | - |  | PL | - | - | - | - |
| 15A0041 | - | + | - |  | PL | - | - | - | - |
| 15A0057 | - | + | - |  | PL | - | - | - | - |
| 15A0060 | - | - | + | Ser | PL | - | - | - | - |
| 15A0061 | + | - | - |  | PL | - | - | - | - |
| 15A0063 | - | - | + | Har/Ser | PL | - | - | - | - |

**Table S2 – *continued***

| Lab ID | Cox-ELISA | Cab-ELISA | Lep  MAT | Lep  Serovar | Organ | mod-ZN Cox-Chl | Cox-qPCR | Chl-qPCR | Lep-qPCR |
| --- | --- | --- | --- | --- | --- | --- | --- | --- | --- |
| 15A0066 | - | + | - |  | PL | - | - | - | - |
| 15A0068 | - | + | - |  | PL | - | - | + (33.52) | - |
| 15A0070 | + | + | - |  | AC | - | - | - | - |
|  |  |  |  |  | PL | - | - | - | - |
| 15A0074 | - | S | - |  | PL | - | - | - | - |
| 15A0076 | - | - | - |  | PL | - | - | + (33.30) | - |
| 15A0077 | - | - | - |  | PL | - | - | - | - |
| 15A0078 | - | - | - |  | PL | - | - | + (33.95) | - |
| 15A0079 | - | - | - |  | PL | - | - | + (33.56) | - |
| 15A0080 | - | + | - |  | PL | - | - | + (34.44) | - |
| 15A0081 | - | - | - |  | PL | - | - | - | - |
| 15A0082 | - | + | + | Har/Ser | PL | - | - | + (34.02) | - |
| 15A0083 | - | S | - |  | PL | - | - | - | - |
| 15A0084 | - | + | - |  | PL | - | - | - | - |
| 15A0086 | - | - | + | Har/Bal | PL | - | + (30.92) | - | - |
| 15A0087 | - | S | - |  | PL | - | + (33.31) | + (29.10) | - |
| 15A0089 | - | - | - |  | PL | - | - | - | - |
| 15A0091 | + | - | - |  | PL | - | - | + (32.93) | - |
| 15A0092 | - | S | - |  | PL | - | - | + (32.05) | + (31.41) |
| 15A0093 | - | + | + | Aus/Aut/Bra | PL | - | - | + (34.62) | - |
| 15A0095 | - | - | - |  | PL | - | - | - | - |
| 15A0096 | + | - | - |  | AC | - | - | + (32.76) | - |
|  |  |  |  |  | PL | - | - | + (34.53) | - |
| 15A0098 | - | + | - |  | PL | - | + (28.38) | - | - |
| 15A0099 | - | - | - |  | PL | - | + (28.78) | + (33.20) | - |
| 15A0101 | - | - | - |  | PL | - | + (31.84) | - | - |
| 15A0103 | - | + | + | Har | PL | - | - | - | - |
| 15A0104 | + | - | - |  | PL | - | - | + (35.21) | - |
| 15A0105 | - | + | - |  | PL | - | - | - | - |
| 15A0106 | - | - | - |  | PL | - | - | - | - |
| 15A0107 | + | - | + | Har/Ser | PL | - | + (31.49) | + (34.11) | + (38.46) |
| 15A0110 | - | + | - |  | PL | - | - | - | - |
| 15A0111 | - | + | - |  | PL | - | - | + (34.09) | - |
| 15A0112 | - | + | + | Har/Ser | PL | - | - | - | - |
| 15A0113 | - | - | - |  | PL | - | - | + (35.57) | - |
| 15A0114 | - | + | + | Har/Ser | PL | - | - | + (35.05) | - |
| 15A0115 | - | - | - |  | PL | - | - | - | - |
| 15A0116 | - | - | - |  | PL | - | - | - | - |
| 15A0117 | - | - | - |  | PL | - | - | + (34.69) | - |
| 15A0118 | - | - | - |  | PL | - | - | + (34.41) | - |
| 15A0121 | - | + | - |  | PL | - | - | + (34.52) | - |
| 15A0122 | - | - | + | Har | PL | - | - | + (35.71) | - |
| 15A0125 | - | - | - |  | PL | - | - | - | - |
| 15A0126 | - | + | - |  | AC | - | - | + (31.84) | - |
|  |  |  |  |  | PL | - | - | - | - |

**Table S2 – *continued***

| Lab ID | Cox-ELISA | Cab-ELISA | Lep  MAT | Lep  Serovar | Organ | mod-ZN Cox-Chl | Cox-qPCR | Chl-qPCR | Lep-qPCR |
| --- | --- | --- | --- | --- | --- | --- | --- | --- | --- |
| 15A0127 | - | S | + | Har | PL | - | - | - | + (38.52) |
| 15A0128 | - | + | - |  | PL | - | - | - | - |
| 15A0129 | - | - | - |  | PL | - | - | + (35.14) | - |
| 15A0130 | - | - | - |  | PL | - | - | - | - |
| 15A0132 | - | - | - |  | PL | - | - | - | - |
| 15A0133 | - | - | - |  | PL | - | - | - | - |
| 15A0134 | - | + | - |  | PL | - | - | - | - |
| 15A0135 | - | - | + | Har | PL | - | - | - | - |
| 15A0136 | - | - | - |  | PL | - | - | - | - |
| 15A0137 | - | - | + | Har/Pom | PL | - | - | + (31.34) | - |
| 15A0138 | - | - | - |  | PL | - | - | - | - |
| 15A0139 | - | - | - |  | PL | - | - | - | - |
| 15A0145 | - | + | - |  | PL | - | - | - | - |
| 15A0146 | - | + | + | Har/Ser | PL | - | - | - | - |
| 15A0147 | - | + | + | Bal | PL | - | - | - | - |
| 15A0148 | - | - | - |  | PL | + | - | + (33.14) | - |
| 15A0149 | - | + | + | Har/Ser | PL | + | - | - | - |
| 15A0150 | - | + | - |  | PL | - | - | - | - |
| 15A0151 | - | - | - |  | PL | - | - | - | - |
| 15A0153 | + | - | - |  | PL | - | - | - | - |
| 15A0154 | - | + | - |  | PL | - | - | - | - |
| 15A0155 | - | + | - |  | PL | - | - | + (34.46) | - |
| 15A0156 | - | + | - |  | PL | - | - | - | - |
| 15A0157 | - | - | + | Har/Ser | PL | - | - | - | - |
| 15A0158 | + | - | - |  | PL | - | - | - | - |
| 15A0159 | - | - | - |  | PL | - | - | - | - |
| 15A0160 | - | - | - |  | PL | - | - | + (34.26) | - |
| 15A0162 | - | S | + | Ser/Har | PL | - | - | - | - |
| 15A0163 | - | + | - |  | PL | - | - | - | - |
| 15A0166 | - | + | - |  | AC | - | - | - | - |
|  |  |  |  |  | PL | - | - | - | - |
| 15A0167 | - | S | + | Har | PL | - | - | - | - |
| 15A0169 | - | - | - |  | PL | - | - | - | - |
| 15A0170 | - | S | - |  | PL | - | - | - | - |
| 15A0171 | - | - | + | Ict/Bal | PL | - | - | - | + (36.23) |
| 15A0172 | - | + | - |  | PL | - | - | + (35.35) | - |
| Total (% of cases) | **15.9** | **38.5** | **21.4** |  |  | **4** | **12.1** | **16.9** | **5.6** |
